# Supplementary material for: A Novel P/N/Si-Containing Vanillin-Based Compound for a Flame-Retardant, Tough Yet Strong Epoxy Thermoset
Source: Polymers (Basel). 2023 May 19;15(10):2384. doi: 10.3390/polym15102384 (PMC10222837; doi:10.3390/polym15102384)
Supplement: Supplementary file 1 [file polymers-15-02384-s001.zip › polymers-2382161.supplementary.conv.docx]

Supporting information

A Novel P/N/Si-Containing Vanillin-Based Compound for a Flame-Retardant, Tough Yet Strong Epoxy Thermoset

Synthesis of VSi-CHO

4,4'-((diphenylsilanediyl)bis(oxy))bis(3-methoxybenzaldehyde), abbreviated as VSi-CHO, is synthesized following the procedure as reported in a previous study. Firstly, 9.129 g (0.06 mol) of vanillin, 5.6 ml (0.0 6ml) of triethylamine, and 100 ml of dichloromethane are added together to a 250 mL three necked flask equipped with a magnetic stirrer, reflux condenser, and nitrogen inlet, and stir evenly under ice bath conditions. Then, 30 ml of dichloromethane dissolved in 7.596 g (0.03 mol) of dichlorodiphenylsilane is slowly dropped into the flask. After the addition is completed, the reaction mixture is heated to reflux temperature and stirred overnight in a nitrogen atmosphere. As the reaction finishes, the produced triethylamine hydrochloride is filtered, and the filtrate is subsequently washed 3~5 times with deionized water and dried over anhydrous magnesium sulfate. At the end, after removing dichloromethane through rotary evaporation and vacuum drying at room temperature for 12 hours, a yellow oily liquid product VSi-CHO is obtained with a yield of 90.8%.


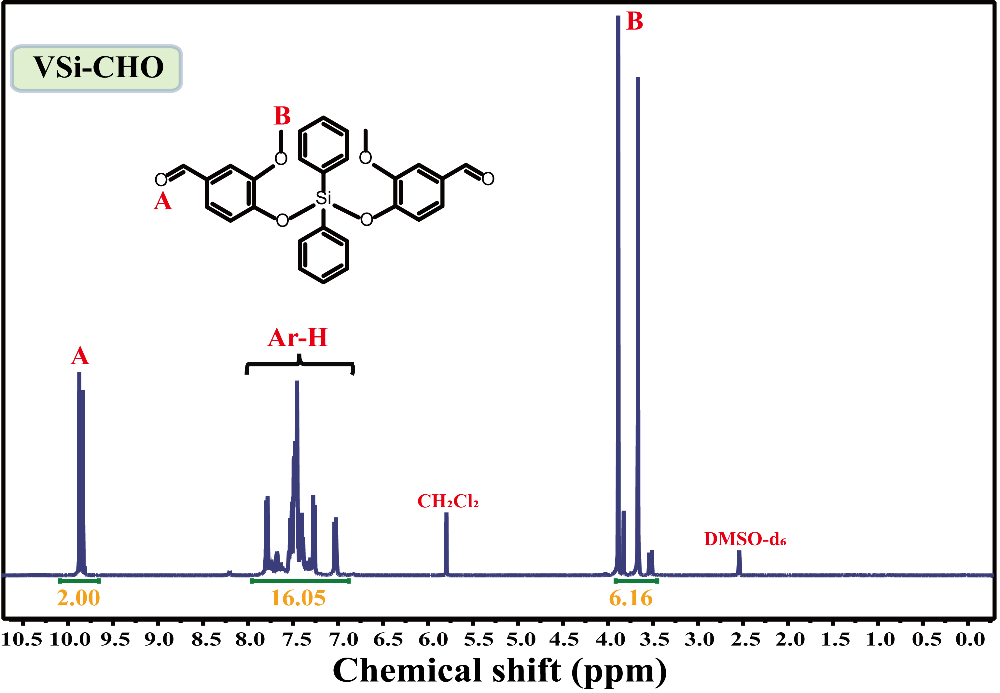


**Figure S1.** ^1^H NMR spectrum of VSi-CHO.


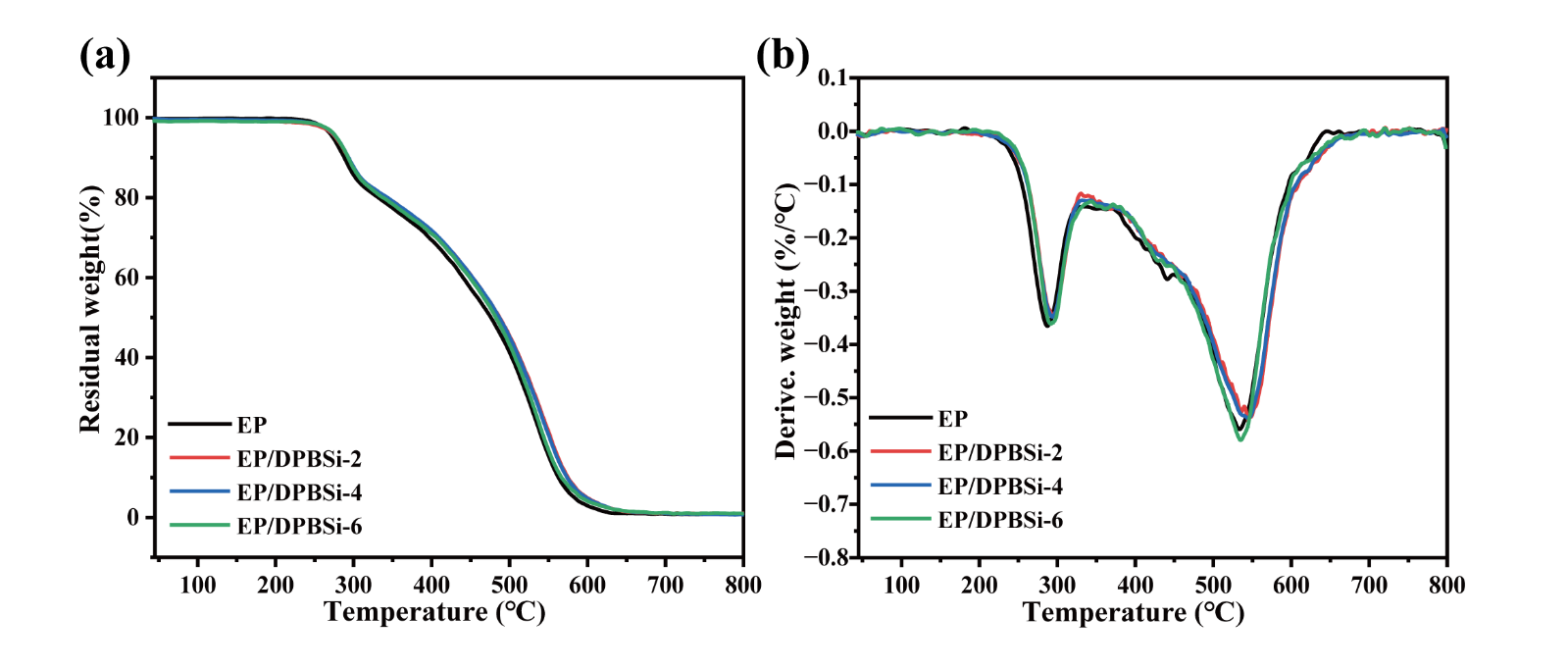


**Figure S2.** TGA(a) and DTG(b) curves of EP and EP composites in air atmosphere.

**Table S1.** TG test data of EP and EP composites under air atmosphere.

| **Samples** | **T_5%_ (℃)** | **T_max1_ (℃)** | **T_max2_(℃)** | **Char yield at 800 °C (%)** |
| --- | --- | --- | --- | --- |
| EP | 273.1 | 287.1 | 534.1 | 0.71 |
| EP/DPBSi-2 | 277.5 | 294.5 | 544.5 | 1.02 |
| EP/DPBSi-4 | 278.2 | 293.2 | 545.2 | 1.01 |
| EP/DPBSi-6 | 278.1 | 291.1 | 534.1 | 1.02 |

*Tmax1: Temperature corresponding to the first maximum decomposition rate

*Tmax2: Temperature corresponding to the second maximum decomposition rate


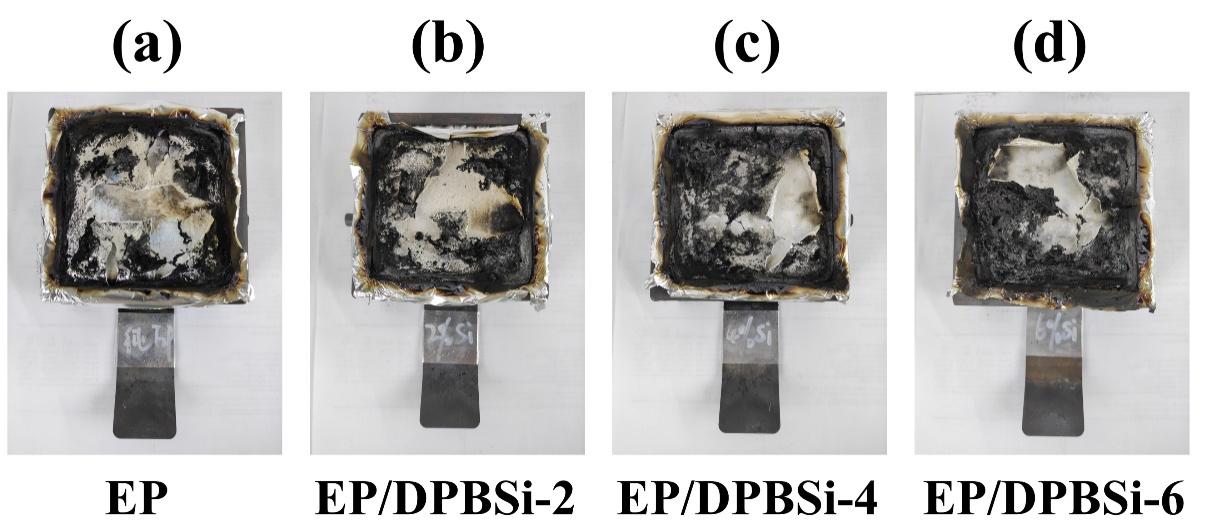


**Figure S3.** Digital photos of EP and EP/DPBSi composites after CCT.


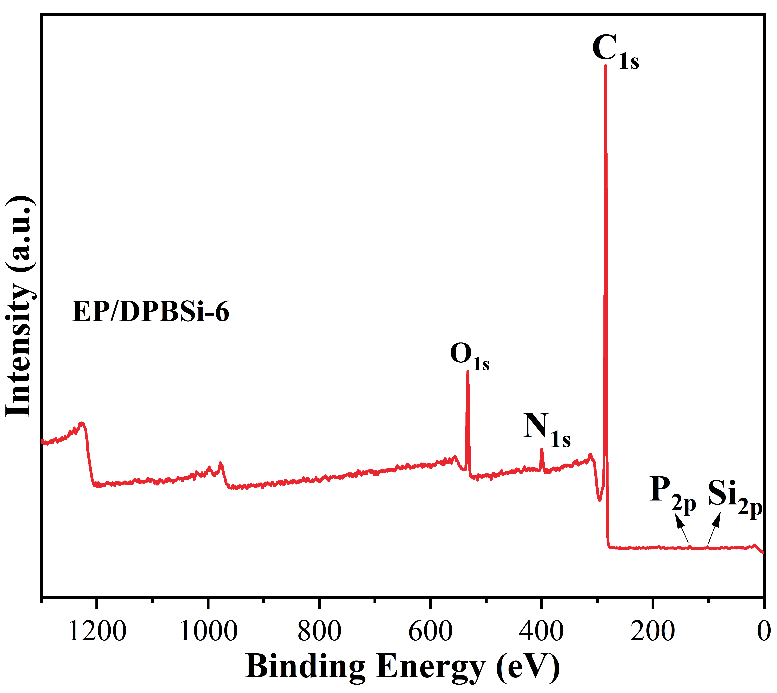


**Figure S4.** XPS spectrum of the char residue of EP/DPBSi-6.


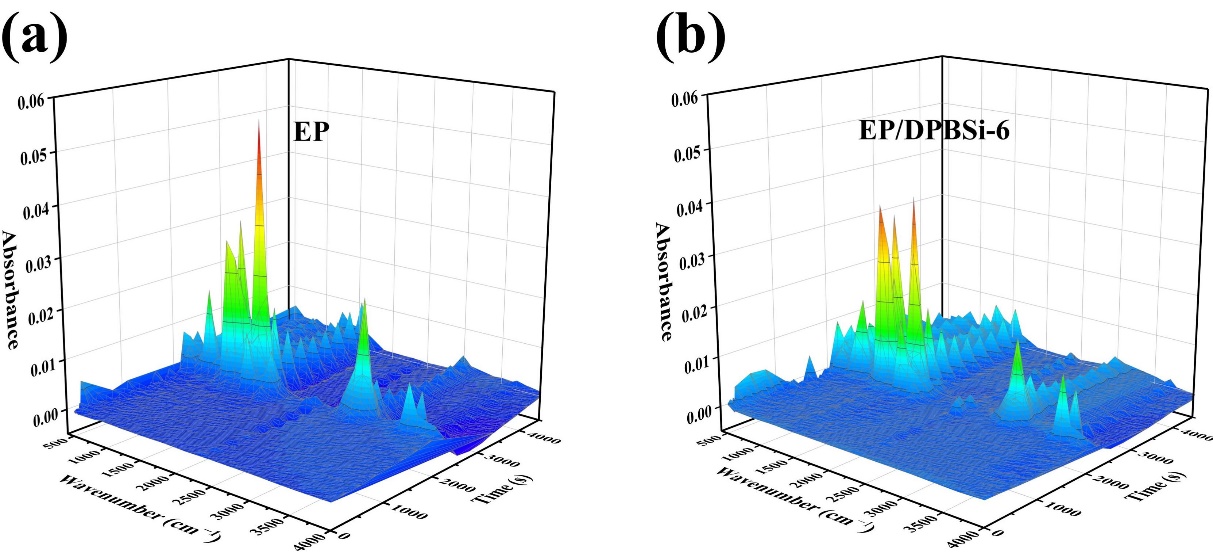


**Figure S5.** TG-FTIR 3D images of EP and EP/DPBSi-6 composites under nitrogen atmosphere.
